# Effect of Laser on the Interface and Thermal Conductivity of Metallized Diamond/Cu Composite Coatings Deposited by Supersonic Laser Deposition

**DOI:** 10.3390/ma17215174

**Published:** 2024-10-24

**Authors:** Yiyun Chen, Qunli Zhang, Bo Li, Zhijun Chen, Shaowu Liu, Xiaofei Ma, Szymon Tofil, Jianhua Yao

**Affiliations:** 1 Institute of Laser Advanced Manufacturing, Zhejiang University of Technology, No. 288 Liuhe Road, Hangzhou 310023, China; 1112102010@zjut.edu.cn (Y.C.); libo1011@zjut.edu.cn (B.L.); roll@zjut.edu.cn (Z.C.); shaowuliu@zjut.edu.cn (S.L.); lam@zjut.edu.cn (J.Y.); 2 College of Mechanical Engineering, Zhejiang University of Technology, No. 288 Liuhe Road, Hangzhou 310023, China; 3 Key Laboratory of Special Purpose Equipment and Advanced Processing Technology, Ministry of Education and Zhejiang Province, Zhejiang University of Technology, No. 288 Liuhe Road, Hangzhou 310023, China; 4 Hangzhou Turbine Power Group Co., Ltd., No. 1188 Dongxin Road, Hangzhou 310015, China; maxf@htc.cn; 5 Centre for Laser Technologies of Metals, Department of Mechatronics and Machine Building, Kielce University of Technology, al. Tysiąclecia Państwa Polskiego 7, 25-314 Kielce, Poland; tofil@tu.kielce.pl

**Keywords:** supersonic laser deposition, metallized diamond, thermal conductivity, adiabatic shear instability, interface bonding

## Abstract

To achieve the rapid heat dissipation of components in the industrial field, the heat dissipation coating is prepared on the surface, which is conducive to improving the service life of the parts and greatly reducing the industrial costs. In this paper, metallized diamond/Cu composite coatings were fabricated on 1060Al substrate by supersonic laser deposition. The composite coatings were prepared at a nitrogen pressure of 3.0 MPa, a scanning speed of 10 mm/s, and a 1060 nm semiconductor coupled fiber laser with different laser power. The research results show that the laser power affects the interface bonding by affecting the temperature of adiabatic shear instability during particle impact. The metallized diamond forms a good bonding at the interface through the plastic deformation of the Cu matrix. Appropriate parameters ensure that the jet does not affect the subsequent particle deposition and build a good heat transfer bridge to elevate the heat transfer efficiency. The coating prepared at a laser power of 1000 W has the highest thermal diffusion coefficient of 89.3 mm^2^/s and thermal conductivity of 313.72 W/(m·K), which is 8.92% higher compared to the coating prepared without laser. Experiments with thermal imaging have also demonstrated that the coating at optimal parameter transferred heat faster. Our research provides a technical guidance for rapid preparation of high-quality heat dissipation coatings in industry.

## 1. Introduction

As industry progresses, many production components are required to serve in extreme environments, ranging from gas turbines, aero-engines, and fan bearings to high-voltage contact switches, chip cooling, and other fields. The further improvement in performance has also challenged the heat dissipation efficiency of the parts, which makes the efficient passive heat dissipation coatings become a hot research topic [1,2,3]. In the early days, a series of metal-based materials such as Cu-Mo, Cu-W, Al/Si, Al/SiC, and ceramic-based materials represented by AlN and BeO were used as heat dissipation materials, but their thermal conductivity has been unable to meet the existing heat dissipation requirements [4,5,6]. Cu has a good thermal conductivity (400 W/(m·K)), the second highest among metals and favorable plasticity, which makes it appropriate for coating design. Diamond possesses a thermal conductivity of nearly 2200 W/(m·K), but its hardness and high cost alone limit its applications in production. Diamond/Cu composites have the advantages of an adjustable coefficient of thermal expansion and good thermal conductivity up to more than 900 W/(m·K) [7]. More and more research is devoted to the development of the application potential of such composites. In addition, the natural non-wettability of diamond and Cu makes poor interfacial bonding, which seriously affects the interface thermal transfer, which can be elevated by the metallization of the diamond [8,9,10].

Conventional techniques for the preparation of diamond/Cu composites include plasma sintering [11], hot press sintering [12], gas pressure infiltration [7], cold spraying [13,14,15], and electrochemical plating [16,17,18]. Sintering and infiltration methods require the use of molds, and the composites prepared have good thermal conductivity. Sun et al. [12] obtained diamond/Cu composites with a thermal conductivity of 638 W/(m·K) by hot-press sintering. The thermal conductivity of diamond/Cu composites prepared by high temperature and high pressure method reaches 900 W/(m·K) [19]. However, there are technological limitations when repairing existing parts or when coatings need to be prepared for the surfaces of certain components. Spraying and electrochemical plating are feasible for the preparation of coatings, but electrochemical plating makes it difficult to achieve specific areas and suffers from weak bonding. There are also contamination problems with the plating solutions used in electroplating technology. The time cost of CVD diamond composite coating is high, and the thickness of the coating is also limited [20]. Cold spray achieves the coating deposition or repair in a local area through the plastic deformation of the particle and formation of metallurgical bonding at the interface through adiabatic shear instability. Its main feature is that the deposition temperature is low to minimize the oxidation and melting problems arising from the high-temperature preparation technology [21]. Its low temperature characteristics can prevent the occurrence of diamond graphitization and Cu oxidation. However, the development of cold-spraying technology faces many challenges, such as the low deposition efficiency of ceramic particles, poor interface bonding, and micro defects [22,23,24,25].

Supersonic laser deposition (SLD) technology couples a laser system with the cold spray technology, which heats the sprayed powder, substrate, and deposited coating in real time during the spraying process. Through the coupling of thermal energy and kinetic energy, the high-quality and efficient deposition of the coating is achieved. SLD overcomes the problems associated with cold spraying and allows for the efficient and fast preparation of the high-quality coatings without dimensional constraints. It has been successfully applied to the preparation of Mo [26], high entropy alloy [27], Inconel 625 alloy [28], WC/Stellite-6 [29], oxide dispersion strengthened alloy [30], etc.

Relevant studies have shown that laser irradiation can energize particles and reduce their critical deposition rate, facilitating the deposition of powders by plastic deformation at relatively low gas-injection rates and increasing the range of machinable materials. The laser can enhance the softening effect of the substrate and improve the interfacial bond strength. However, too much laser power creates a large temperature gradient, which will cause the phenomenon of thermal stress cracking [26]. Laser can reduce the porosity of the material while improving the homogeneity of the second phase [31]. Previous research has focused on the study of fatigue performance and wear resistance, but there has been less study of the thermal properties of composite coatings prepared by SLD [8,28,31,32].

There is a lack of systematic research on the heat-transfer performance of metallized diamond/Cu composite coating deposited by supersonic laser deposition. In this paper, the influence of laser power on the deposition interface characteristics of metallized diamond and the heat-transfer performance of the coating is revealed by changing the laser power. The effect of laser power on the volume content of diamond in the coating, density, hardness, and other basic physical properties was studied. The mechanism of laser irradiation on the micro-interface is revealed. The heat dissipation of the composite coatings was characterized by thermal imaging technology.

## 2. Materials and Methods

### 2.1. Materials and Preparation Methods

**Figure 1:**
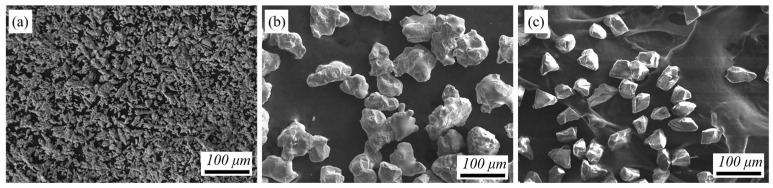
The morphology of the original powder: (**a**) Cu, (**b**) Cr, and (**c**) diamond.

The experimental raw materials include irregular Cu powder with the size of 15~53 μm (Zhongke Yanuo Technology Co., LTD., Beijing, China) and 35~42 μm irregular diamond powder (Zhongyuan Superhard Company, Shangqiu, Henan, China). Figure 1 shows the morphology of the experimental powders. Chemical composition of experimental powders and Al substrate is given in Table 1. Chromium carbide was plated on the surface of diamond by salt bath method at 900 °C for 1 h. The 1060Al substrate was sandblasted with 24 μm corundum and ultrasonic-cleaned with ethanol for 30 min. The 50 vol% diamond composite powder was mechanically mixed with Cu before spraying, and the Cr-coated diamond/Cu composite coating was deposited on Al substrate by supersonic laser deposition technology [33].

**Figure 2:**
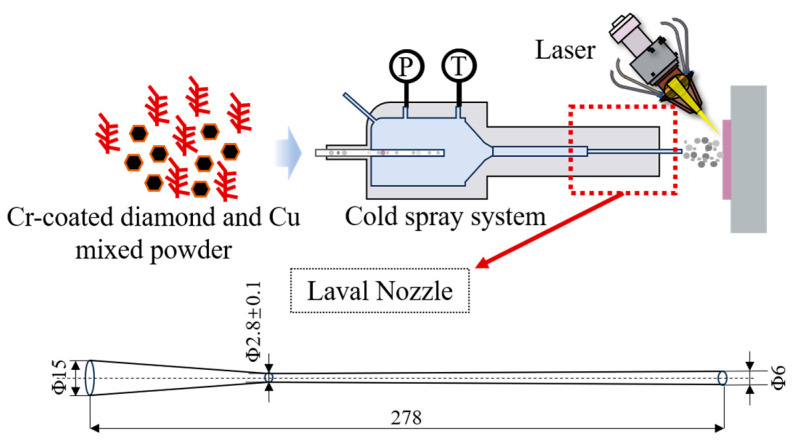
The schematic diagram of SLD and the nozzle parameters (unit: mm).

The supersonic laser deposition system mainly consists of a PCS-1000 cold spray system and a 1060 nm semiconductor coupled fiber laser system (LDF6000—100 VGP, Laserline, Koblenz, Germany). Figure 2 shows the schematic diagram of experimental supersonic laser deposition. The length of Laval nozzle is 278.0 mm, the throat diameter is 2.8 ± 0.1 m, and the outlet diameter is 6.0 mm.

Through preliminary experiments, we found that under the laser power of 2000 W, Al substrate would melt. And high laser power would also lead to diamond graphitization and local cracking problems. Referring to the team’s previous research [8,32], 0 W, 500 W, 1000 W and 1500 W were selected as suitable range of process parameters. The incident angle of the laser is 30° with the spray gun, and the plane formed by the two is perpendicular to the sprayed substrate. All samples were prepared at a spraying distance of 30.0 mm, a nitrogen pressure of 3.0 MPa, and a scanning speed of 10 mm/s. To facilitate the discussion, the samples prepared at different laser powers were set up with the abbreviation. The abbreviation “P” indicates the laser power of the deposited composite coating, while the subscripts “0”, “500”, “1000”, and “1500” indicate the value of the laser power.

### 2.2. Characterization Methods

The phase composition was detected by X-ray diffraction (D8 Advance, Bruker, Billerica, MA, USA) with a Cu source (λ = 1.542 A, 40 mA, 40 kV). The microstructure of the diamond distributions and interface between diamond and Cu were characterized by scanning electron microscopy (SEM, EVO 18, Carl Zeiss, Stuttgart, Germany). The elemental composition of the samples was determined by an EDS (Nano X-flash Detector 5010, Bruker). The microhardness was tested by HPV-2 automatic Vickers hardness tester (Shimadzu Company, Nakao-ku, Kyoto, Japan). The loading time was 10 s and the test load was 0.1 kgf.

### 2.3. Thermal Properties Test

The thermal diffusivity α of the composite coating (10 * 10 * 3 mm^3^) at room temperature was examined by a laser thermal diffusiometer (NETZSCH LFA467, Selb, Bavaria, Germany). Before testing, the surface of the composite coating was coated with graphite to reduce heat loss and improve test accuracy. Each sample was tested 3 times and averaged to prevent experimental contingency. The calculation formula of thermal conductivity of composite coating is as follows: λ = α * ρ * C_p_. Where λ is the thermal conductivity (W/(m∙K)), and ρ (kg/m^3^) is the density measured based on Archimedes’ principle, α (m^2^/s) is the thermal diffusion coefficient and C_p_ (J/(kg∙K)) is the specific heat capacity, which is calibrated by the standard sample (graphite) through the LFA equipment.

The same sample size as for the thermal conductivity tests was used for experiments on heat dissipation applications. A thermal imager (FLIR A615, Wilsonville, OR, USA) was employed to record the heating process of composite coating. The samples were placed on a thermostatically heated table and a screen was used around them to minimize air flow.

## 3. Results

### 3.1. Pretreatment of Diamond Metallized Powder

**Figure 3:**
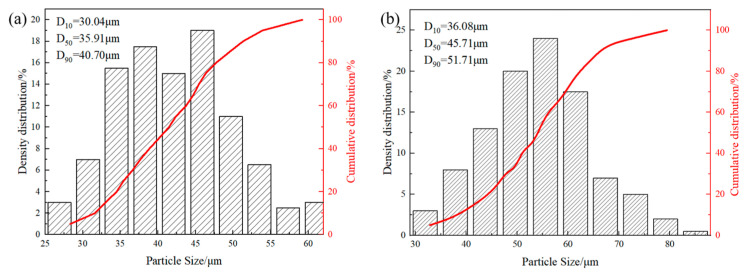
The particle size distribution before and after the salt bath: (**a**) original diamond and (**b**) metallized diamond.

The interfacial bonding between diamond and Cu can be elevated by matrix alloying and diamond metallization, so as to reduce the interface acoustic impedance. In this paper, a layer of chromium carbide with uniform thickness was prepared on the surface of diamond to improve interfacial heat transfer efficiency, which was used to prepare metallized diamond/Cu composite coating by SLD. Figure 3 shows the particle size distribution before and after the salt bath. Figure 3a shows that the average diamond particle size is at 36 µm, while the metallized diamond particle size is 46 µm in Figure 3b, estimating the coating thickness to be around 5 µm.

**Figure 4:**
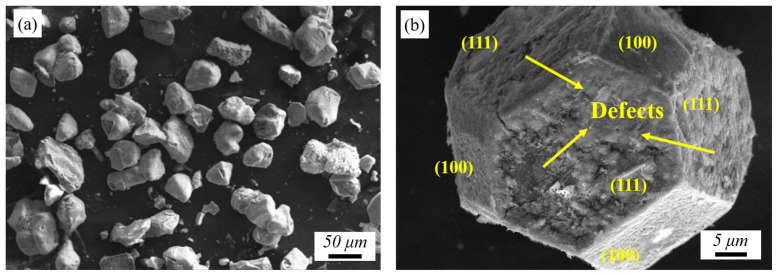
The morphology of diamond after salt bath reaction: (**a**) metallized diamonds and (**b**) a regular dodecahedral diamond.

Figure 4 shows the morphology of diamond after salt bath reaction. Figure 4a shows that the diamond surface is covered with transition layer. Figure 4b shows the morphology of a regular dodecahedral diamond found in irregular powder. It can be observed that there are defects in the growth of (111) crystal face, while the growth of (100) crystal face is relatively complete, which is linked to the different surface energies of different crystal faces. The surface energy of (111) crystal surface is 3.387 J/m^2^, while the surface energy of (100) crystal surface is 9.2 J/m^2^ [10]. The high-energy surface is more likely to react into carbides. In the process of supersonic deposition of metallized diamond, the transition layer can provide buffer for the impact process of diamond [34], reduce the risk of brittle breakage of diamond, and protect the diamond from graphitization due to high temperature during deposition [35].

### 3.2. Effect of Laser Power on the Microstructure of Composite Coatings

**Figure 5:**
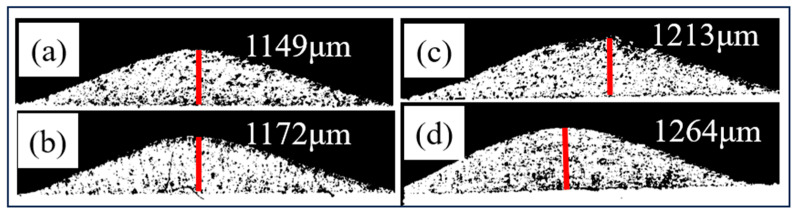
The single-track thickness of the composite coating deposited by SLD under different laser powers: (**a**) P_0_, (**b**) P_500_, (**c**) P_1000_, and (**d**) P_1500_.

Figure 5 shows the single-track thickness of the composite coating deposited by SLD under different laser powers. With other process parameters unchanged, the increase in laser power can promote the increase of coating thickness. The laser mainly affects the diamond content in the coating and the bonding of the micro-interface, which would be discussed later.

**Figure 6:**
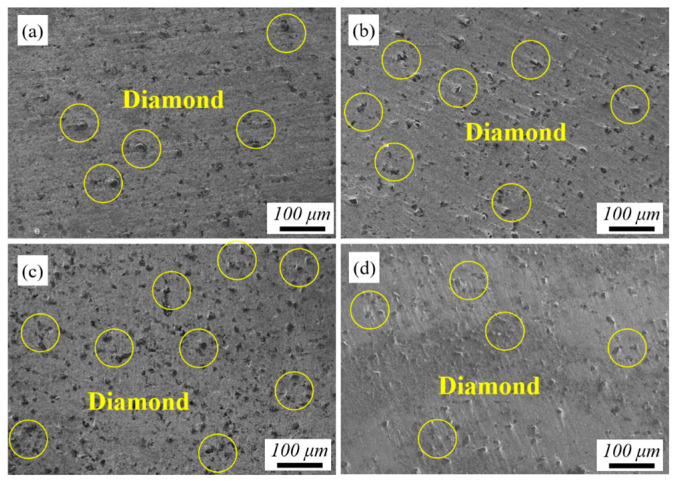
The distribution of diamond in the composite coating deposited by SLD under different laser powers: (**a**) P_0_, (**b**) P_500_, (**c**) P_1000_, and (**d**) P_1500_.

Figure 6 shows the distribution of diamond in the composite coating deposited by SLD at different laser powers. Diamond content is the key factor impacting the TC of composite coating [36]. P_0_ without laser irradiation shows that the diamond content is low and unevenly dispersed in Figure 6a. P_500_ shows that the diamond content is significantly increased and the distribution is more uniform in Figure 6b. Compared to Figure 6b, the diamond content in Figure 6c is higher and the diamonds form a network-like distribution, which is favorable for the formation of heat-transfer pathways. However, when the laser power is 1500 W, the diamond content decreases, and the uniformity becomes worse in Figure 6d. It is mainly due to the fact that the deposition surface undergoes severe plastic deformation or even melting with high laser power, which makes the probability of impact become larger between post-deposited diamonds and deposited diamonds and the rebound leads to the decline in diamond content.

**Figure 7:**
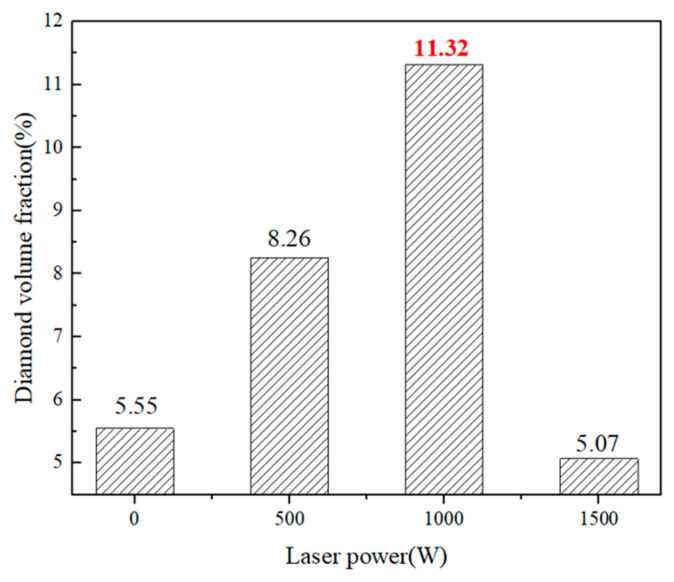
The diamond volume content of the composite coatings prepared under different laser powers. The color red is used to highlight the value.

Figure 7 shows the diamond volume content of the coatings fabricated under different laser powers, which were statistically calculated from the SEM images using image J software (1.53c, National Institutes of Health, Bethesda, MD, USA). The variation trend of diamond volume content against laser power is consistent with Figure 6. Too-high or too-low laser power affects the diamond deposition efficiency. At 1000 W, the diamond volume content fraction reaches up to 11.32%, which is the largest. At a low laser power, the degree of the surface plastic deformation of the diamond on impact is weak, making it difficult to deposit with a large critical velocity. A high laser power causes excessive softening and the diamonds to collide with each other and break and rebound [14],which also causes risks such as oxidation and graphitization [37]. Due to the brittle nature of diamond, deposition hardly occurs under conventional conditions. However, the deposition efficiency of diamond is greatly improved by metallization and the plasticity of the Cu matrix under the action of the laser, which provides a guiding idea for the industrial low-cost use of diamond in the preparation of coatings.

**Figure 8:**
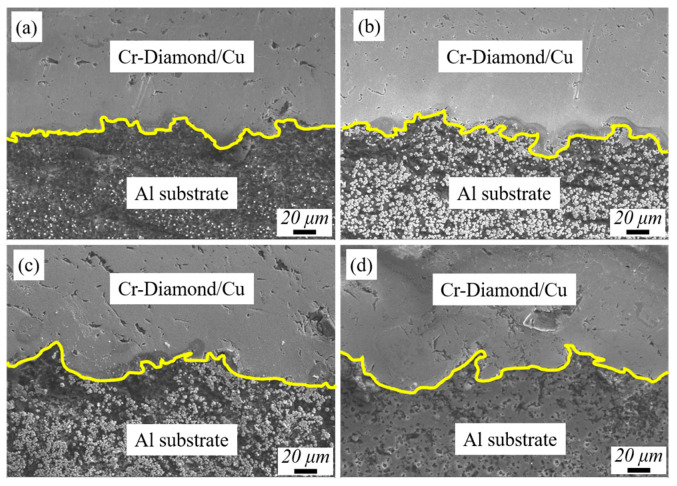
The interface between the coating and 1060 Al substrate fabricated under different laser powers: (**a**) P_0_, (**b**) P_500_, (**c**) P_1000_, and (**d**) P_1500_.

Figure 8 shows the interface between the Cr-coated diamond/Cu composite coating and 1060Al substrate prepared under different laser powers. As the laser power rises, the degree of bonding with Al substrate interface increases, mainly judging from the degree of deformation at the interface. The mutual chimeric degree of particle impact is increased, which is due to the continuous laser irradiation. The Al substrate is violently deformed at the interface when the particles are impacted, forming a vortex-like mechanical chimeric. In Figure 8c, the yellow interfacial demarcation of the P_1000_ contains both large and small deformation chimeras which possesses better interfacial bonding.

**Figure 9:**
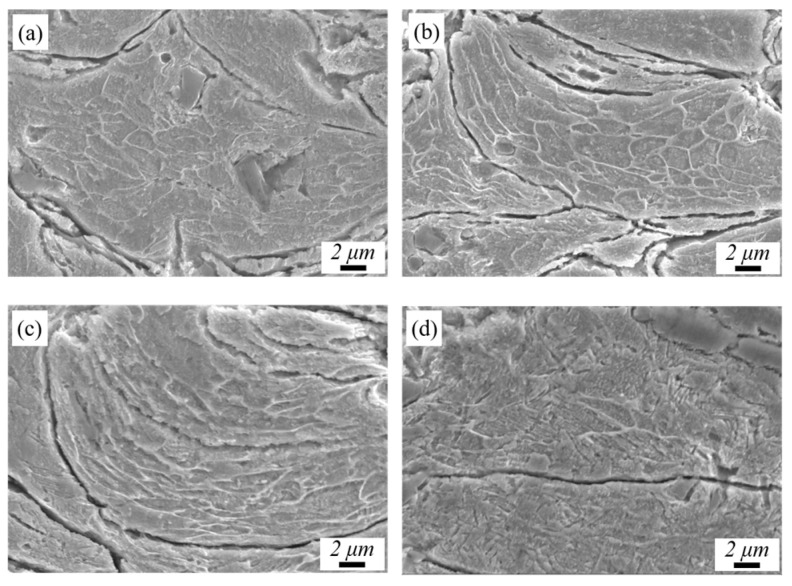
The plastic deformation of the Cu under different laser powers: (**a**) P_0_, (**b**) P_500_, (**c**) P_1000_, and (**d**) P_1500_.

Figure 9 shows the plastic deformation of the Cu matrix under different laser powers. The Cu matrix undergoes different degrees of plastic deformation during the impact process due to the different degrees of laser thermal effect. As can be seen in Figure 9a, the grains inside the cold-sprayed middle particles basically maintain their original morphology, and the flattening tendency only occurs at the edges of the particles. In Figure 9b, the grains at the interface of P_500_ are elongated, while the middle parts still maintain their original morphology. In Figure 9c, with a laser power of 1000 W, the plastic deformation of the grains is characterized by impact jets, and the whole grain has been severely deformed, while the internal grains are basically all flattened. The cold-sprayed Cu coating is mainly mechanically bonded and there is a small amount of metallurgical bonding [38]. In Figure 9c, the particles are tightly bonded at the interface, and metallurgical bonding may occur. When the laser power is 1500 W, in Figure 9d, the micro-fusion of the internal particles occurs, leading to the gradual blurring of the interface of the grains, and the Cu would be oxidized, affecting the coating performance.

**Figure 10:**
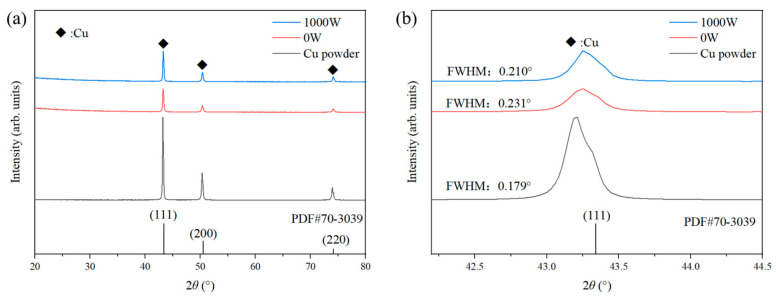
The XRD patterns of Cu powder, P_0_, and P_1000_: (**a**) all peaks and (**b**) half peak width of (111).

Figure 10 shows the XRD analysis of Cu powder, P_0_, and P_1000_. Dynamic recrystallization is the stress concentration caused by particle impact, resulting in plastic deformation to form nanocrystals. Under the action of laser, the shaping deformation of copper base is more likely to occur, and the internal drive of recrystallization is greater, so that the crystallinity is increased compared with cold spraying. Compared with the half peak width of the enlarged (111) peak, the crystallinity of the powder is high, and the amorphous grains occur after the formation of the coating due to the adiabatic shear instability mechanism caused by the impact [39].

**Figure 11:**
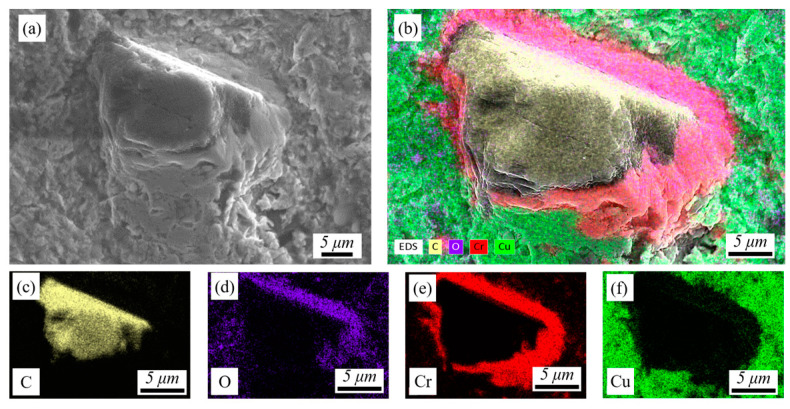
SEM and EDS at the interface of diamond and Cu in P_1000_: (**a**) the morphology of the interface, (**b**) Mapping of all elements and (**c**–**f**) Mapping of the element: C, O, Cr and Cu.

Figure 11 shows SEM and EDS at the interface of diamond and Cu in P_1000_. Figure 11a shows that diamond is tightly embedded in the Cu matrix, and the diamond forms a bond with the Cu through the interlayer. Diamond is chemically bonded to Cr, while Cr-coated diamond is mechanically and partially metallurgically bonded to the Cu matrix during the deposition process. The Cr and chromium carbide interlayer improves the transport efficiency by reducing the phonon scattering at the interface, thereby increasing the thermal conductivity of the interface.

**Figure 12:**
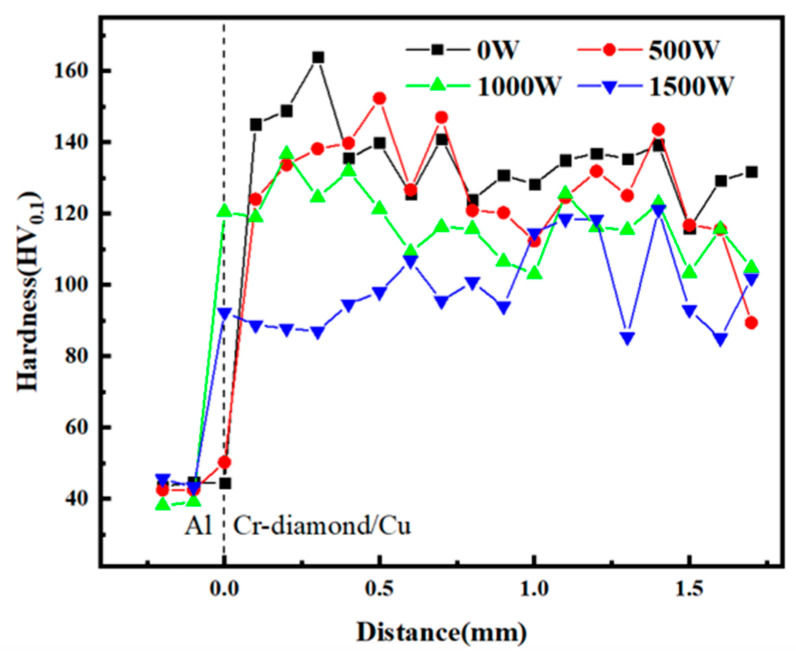
The hardness of composite coatings under different laser powers.

The influence of laser power on coating hardness is shown in the Figure 12. As laser power rises, the hardness of the composite coating gradually decreases, which is chiefly owing to the removal of work hardening by laser thermal effect. The average hardness of P_1000_ drops slightly to a value of 120 HV, whereas a drop in hardness of P_1500_ to below 100 HV would be unfavorable to the functioning of the coating. The hardness test point is on the copper base, so it mainly reflects the hardness of the matrix. Because the diamond only breaks or is completely deposited during the impact, basically, it does not undergo plastic deformation. The diamond hardness is not applicable to the study of the change in the hardness of the deposited coating.

### 3.3. Influence of Laser on Thermal Conductivity

**Figure 13:**
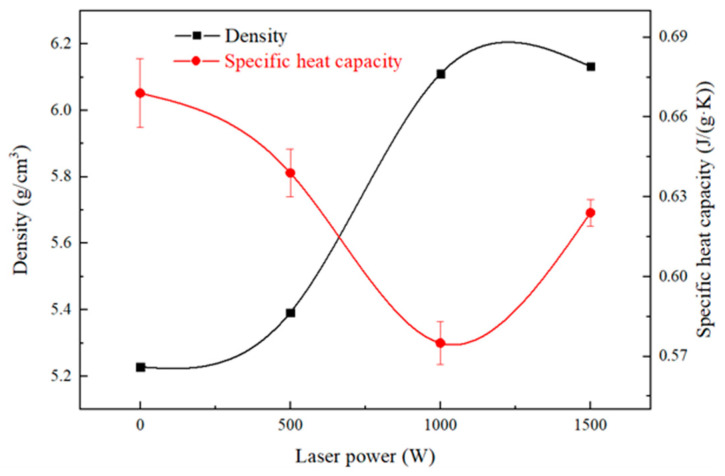
The tendency of density and specific heat capacity on laser power.

The thermal conductivity of composites depends on many factors, which are chiefly influenced by the properties and content of the second phase, the matrix properties and microstructure, and the interfacial thermal conductivity. In this paper, the effects of microstructure and interfacial thermal conductance on the thermal conductivity of supersonic laser deposition composite coating are studied. The thermal-physical properties of the coating with the change in laser power are shown in Table 2. The density of the coatings increases as the laser power rises, which shows that laser plays an important role in the densification of the coating. However, from the trend chart of density and laser power in Figure 13, it can be found that the density reaches 6.11 g/cm^3^ when the laser power reaches 1000 W. Further increasing the laser power has a minor effect on density improvement, while the specific heat capacity is the smallest at 1000 W.

**Figure 14:**
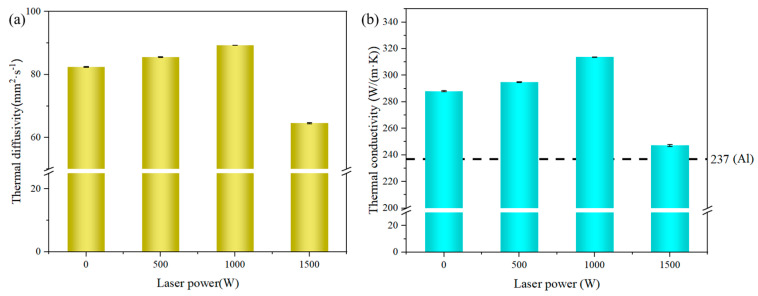
Thermal diffusion coefficient (**a**) and thermal conductivity (**b**) of composite coatings prepared under different laser powers.

Figure 14 shows the thermal diffusion coefficient and thermal conductivity of metallized diamond/Cu composite coatings prepared at different laser powers. P_1000_ has the highest thermal diffusion coefficient of 89.3 mm^2^/s, which is 8.37% higher compared to P_0_. The tendency of the thermal conductivity is similar to that of the thermal diffusion. As the laser power rises, the thermal conductivity of the coatings increases and then decreases in Figure 14b. The highest thermal conductivity is 313.72 W/(m·K), which is 8.92% higher compared to the coating prepared without laser and more than 1.65 times higher than that of the traditional cold-sprayed coating [40]. It is noteworthy that the coating thermal diffusivity and thermal conductivity are the lowest at 1500 W laser power, which may be related to the microstructure of the coating.

**Figure 15:**
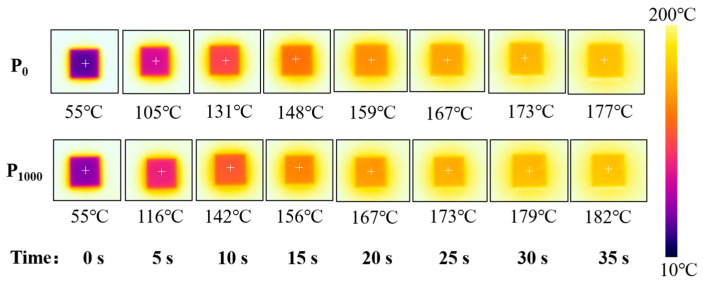
The infrared thermal imaging changes of P_0_ and P_1000_.

**Figure 16:**
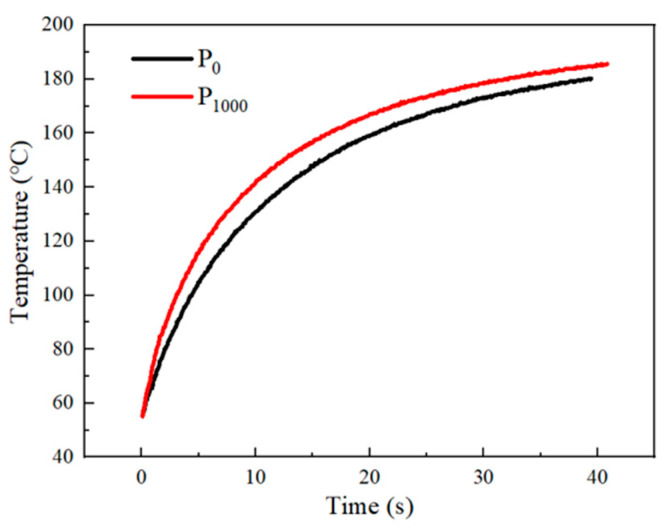
The temperature variation of P_0_ and P_1000_.

Figure 15 shows the infrared thermal imaging changes of P_0_ and P_1000_ on the constant temperature heating table. It can be seen that the temperature of the center point of the surface of the sample without laser rises slowly, while the temperature of the sample prepared under 1000 W laser power rises faster, which is mainly due to the difference in thermal diffusivity between P_0_ and P_1000_. From the thermal diffusivity in Figure 14, it is evident that the thermal diffusivity of the sample prepared by 1000 W laser power is the highest. Therefore, it has the better heat dissipation capacity. Figure 16 shows the heating process of P_0_ and P_1000_. It can be seen that the heat transfer of P_1000_ sample is faster in the same environment, and the temperature is always higher than that of P_0_ before reaching the steady state.

The thermal conductivity mechanism of metallized diamond/Cu composite coating deposited by SLD is mainly caused by microstructure characteristics. Different from the coatings prepared by laser cladding or thermal spraying technology, it is characterized by deposition through plastic deformation. The heat-transfer mechanism between copper particles is the different degree of interface bonding caused by the different flatness of particles under the action of laser. The phonon would generate thermal resistance at the interface during the transmission process, and the higher the flatness, the higher the phonon transmission efficiency. The main mechanism of diamond and Cu transmission is that the metallized transition layer fills the gap between the two, which reduces the acoustic impedance difference between diamond and Cu, reduces phonon scattering, and improves phonon transmission; therefore, it reduces the interface thermal resistance.

## 4. Conclusions

In this paper, the metallized diamond/Cu composite coating was fabricated by supersonic laser deposition. The microstructure and interface at different laser powers were studied. The effect of laser on the thermal conductivity of the coating was explained. The laser irradiation elevated the diamond content in the coating and improved the bonding interface. The laser intensifies the degree of the plastic deformation of the Cu, resulting in the formation of a coating with higher density, reducing the defects at the micro-interface and helping to improve the thermal conductivity. The crystallinity is slightly increased under the action of the laser, which can also contribute to the improvement of the thermal conductivity. The metallized micro-interface improved the non-wettability of diamond and copper under the action of laser, elevated the heat transfer efficiency at the interface, and reduced the degree of work hardening. The coating prepared at a laser power of 1000 W has the highest thermal diffusion coefficient of 89.3 mm^2^/s and thermal conductivity of 313.72 W/(m·K), which is 8.92% higher compared to the coating prepared without laser. The research provides a reliable basis for the rapid preparation of thermal coating without scale under an open environment, and it has great application potential in industrial thermal components and radiators.

## Figures and Tables

**Table 1:** Chemical composition of experimental powders and Al substrate.

**Element**	Cu	Bi	Pb	Sb	Sn	O	Zn	Ni	Fe
**wt.%**	Bal.	≤0.001	≤0.003	≤0.002	≤0.002	≤0.002	≤0.003	≤0.002	≤0.004
**Element**	Al	Fe	Si	Cu	Mg	Zn	Mn	Ti	V
**wt.%**	Bal.	≤0.35	≤0.25	≤0.05	≤0.03	≤0.05	≤0.03	≤0.03	≤0.05
**Element**	Cr	Fe	Si	Al	Cu	C	S		
**wt.%**	Bal.	≤0.40	≤0.30	≤0.30	≤0.04	≤0.02	≤0.02		
**Element**	Diamond	N	P	O	Sr	Ba			
**wt.%**	Bal.	≤0.001	≤0.001	≤0.001	≤0.001	≤0.001			

**Table 2:** The thermal-physical properties of the coatings under different laser powers.

Sample	Density (g/cm^3^)	Coefficient of Thermal Diffusion (mm^2^/s)	Specific Heat Capacity (J/(g·K))	Thermal Conductivity (W/(m·K))
P_0_	5.228	82.361 ± 0.115	0.669 ± 0.013	288.006 ± 0.402
P_500_	5.392	85.554 ± 0.099	0.639 ± 0.009	294.907 ± 0.341
P_1000_	6.110	89.306 ± 0.034	0.575 ± 0.008	313.718 ± 0.119
P_1500_	6.132	64.587 ± 0.188	0.624 ± 0.005	247.150 ± 0.720

## Data Availability

The original contributions presented in the study are included in the article, further inquiries can be directed to the corresponding author.
